# QUANTIFICATION OF PULMONARY PATHOLOGY IN CYSTIC FIBROSIS–COMPARISON BETWEEN DIGITAL CHEST TOMOSYNTHESIS AND COMPUTED TOMOGRAPHY

**DOI:** 10.1093/rpd/ncab017

**Published:** 2021-03-04

**Authors:** C Meltzer, M Gilljam, J Vikgren, R R Norrlund, K Vult von Steyern, M Båth, Å A Johnsson

**Affiliations:** Department of Radiology, Institute of Clinical Sciences, Sahlgrenska Academy, University of Gothenburg, Bruna stråket 11b V 2 SU/Sahlgrenska, 413 45 Gothenburg, Sweden; Department of Radiology and Nuclear Medicine, Oslo University Hospital, Nydalen 0424 Oslo, Norway; Gothenburg CF-Center, Sahlgrenska University Hospital, Gothenburg, Sweden; Department of Respiratory Medicine, Institute of Medicine, Sahlgrenska Academy, University of Gothenburg, Gothenburg Sweden; Department of Radiology, Institute of Clinical Sciences, Sahlgrenska Academy, University of Gothenburg, Bruna stråket 11b V 2 SU/Sahlgrenska, 413 45 Gothenburg, Sweden; Department of Radiology, Sahlgrenska University Hospital, Bruna stråket 11b V 2 SU/Sahlgrenska, 413 45 Gothenburg, Sweden; Department of Radiology, Institute of Clinical Sciences, Sahlgrenska Academy, University of Gothenburg, Bruna stråket 11b V 2 SU/Sahlgrenska, 413 45 Gothenburg, Sweden; Department of Radiology, Sahlgrenska University Hospital, Bruna stråket 11b V 2 SU/Sahlgrenska, 413 45 Gothenburg, Sweden; Center for Medical Imaging and Physiology, Skåne University Hospital, Getingevägen 4, 22185 Lund, Sweden; Department of Medical Physics and Biomedical Engineering, Sahlgrenska University Hospital, Gula stråket 2B, Plan 3, 413 45 Gothenburg, Sweden; Department of Radiation Physics, Sahlgrenska Academy, University of Gothenburg, Gula stråket 2B, Plan 3, 413 45 Gothenburg, Sweden; Department of Radiology, Institute of Clinical Sciences, Sahlgrenska Academy, University of Gothenburg, Bruna stråket 11b V 2 SU/Sahlgrenska, 413 45 Gothenburg, Sweden; Department of Radiology, Sahlgrenska University Hospital, Bruna stråket 11b V 2 SU/Sahlgrenska, 413 45 Gothenburg, Sweden

## Abstract

Purpose: Digital tomosynthesis (DTS) is currently undergoing validation for potential clinical implications. The aim of this study was to investigate the potential for DTS as a low-dose alternative to computed tomography (CT) in imaging of pulmonary pathology in patients with cystic fibrosis (CF). Methods: DTS and CT were performed as part of the routine triannual follow-up in 31 CF patients. Extent of disease was quantified according to modality-specific scoring systems. Statistical analysis included Spearman’s rank correlation coefficient (*r*) and Krippendorff’s alpha (*α*). Major findings: The median effective dose was 0.14 for DTS and 2.68 for CT. Intermodality correlation was very strong for total score and the subscores regarding bronchiectasis and bronchial wall-thickening (*r* = 0.82–0.91, *P* < 0.01). Interobserver reliability was high for total score, bronchiectasis and mucus plugging (*α* = 0.83–0.93) in DTS. Conclusion: Chest tomosynthesis could be a low-dose alternative to CT in quantitative estimation of structural lung disease in CF.

## INTRODUCTION

**Figure 1:**
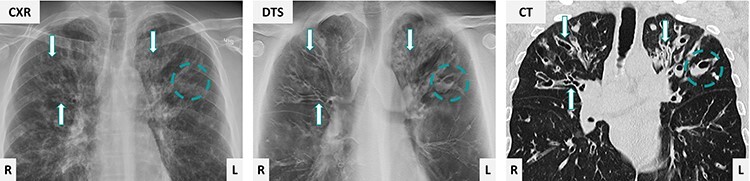
An adult patient with CF examined by CXR, DTS and CT. Pathological changes such as wall-thickened bronchiectasis (arrows) and mucus plugging (circles) are difficult to delineate on CXR because of overlapping anatomy, though clearly visible on DTS and CT.

Digital tomosynthesis (DTS)^(1–3)^ is a relatively new modality based on the same technique as conventional radiography. Visualisation of pathology is superior to chest x-ray (CXR), and at lower cost, reading time and radiation exposure than computed tomography (CT).^(1–4)^ The technique can be accessible by an upgrading of software and hardware, based on the equipment for conventional radiography, including a moving tube, which acquires multiple low-dose projections during an angular movement. Standard protocol for chest imaging is an angular range ± 15° relative to the standard posterior–anterior imaging plane. The effective radiation dose is ~0.13 mSv, compared to 0.07–0.10 mSv for CXR and 1–3 mSv for standard CT.^(5)^ The angular movement enables separation of overlapping anatomy, otherwise superimposed on CXR,^(6)^ and DTS has been reported as a problem solver to inconclusive findings in CXR,^(7,8)^ an imaging example is presented in Figure 1.

Cystic fibrosis (CF)^(9)^ is a congenital disease causing pathological thickening of mucus with impaired clearance of inhaled particles and infectious agents, which leads to obstruction, inflammation and infection. Clinical symptoms range from none to life-threatening, and respiratory failure is the main reason for shortened lifetime expectancy. Patients undergo life-long imaging surveillance of pulmonary pathology from early childhood, and the need for optimization of radiation exposure is essential. CT is considered the reference modality for chest imaging, but contribute to considerable radiation exposure and a high number of incidental findings.^(10,11)^ DTS has been suggested as an alternative to CT, however, impaired visibility of structures in the anterior, posterior and lower parts of the lungs has been reported in patients with CF^(12)^ and studies on nodule detection indicate reduced rates for DTS compared to CT.^(4,13,14)^

Monitoring of pulmonary pathology is central in CF, since early intervention can minimise progressive, irreversible lung damage, and the introduction of the promising but costly CFTR modulator therapies raises an additional need for quantitative evaluation of the effect of treatment. CT imaging can identify patients with substantial and progressive structural changes and still normal or unchanged pulmonary function tests (PFTs).^(15–17)^ Due to radiation concern, CXR is often used in both surveillance and acute exacerbations in CF; however, minor structural changes can be difficult to identify, and the European CF guidelines recommend availability to other modalities such as CT.^(18)^ Magnetic resonance imaging shows promising results,^(19–22)^ however, not yet implemented in clinical routine. The most important changes related to CF in diagnostic imaging involve bronchiectasis and mucus plugging,^(23)^ other typical changes are bronchial wall thickening, airtrapping and consolidation. To meet the need for standardised quantification of the extent of disease modality-specific scoring, systems have been developed.^(16,19,21,24,25)^ Vult von Steyern (VvS) developed a scoring system for DTS in 2012 and presented good agreement with Brasfield score for CXR.^(26)^ A recent study reported good correlation between the tomosynthesis scoring and Brasfield score.^(27)^ However, there are no available studies comparing DTS and CT regarding quantitative surveillance of structural lung disease in CF.

The aim of this study was to investigate DTS in quantification of pulmonary CF-pathology in comparison to CT using modality-specific scoring systems and observers with expert knowledge in the field.

## MATERIAL AND METHODS

### Participants

**Figure 2:**
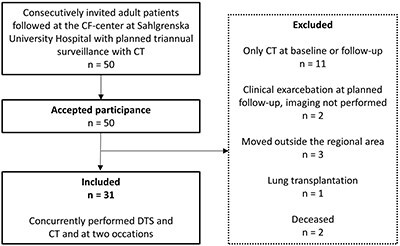
Participant flowchart.

Adult patients followed at (Gothenburg) CF-centre are undergoing routine surveillance with annual CXR and triannual CT, and participants were consecutively invited to this prospective, observational study performed between March 2011 and February 2017. Imaging and PFT were part of the follow-up performed at two occasions separated by 3 years, and the study protocol involved DTS in addition to the routine CT. The study was approved by the regional ethical board and all participants gave their written informed consent. A proportion of the baseline examinations (15/31) were included in a previous study regarding the dependency of anatomical location and observer experience for visualisation of specific structures in DTS,^(12)^ but none of the observers participated in both studies. A participant flowchart is presented in Figure 2.

### Index test

DTS was performed with GE Definium8000 (March 2011–April 2016, *n* = 45) and GE DiscoveryXR656 (May 2016–February 2017, *n* = 17) equipment with the VolumeRAD option (GE Healthcare). A frontal CXR was used as a scout, and imaging parameters for DTS was according to recommended standard settings.^(28)^ Approximately 60 low-dose projections were acquired during a 10s breath hold, in standing position at full inspiration during a caudocranial tube movement ±15° from the standard postero-anterior direction. Tube voltage was 120–125 kV and additional filtration were 3 mm aluminium and 0.1 mm copper. The examination also included a lateral CXR. The low-dose projections were reconstructed to ~60 section images in the coronal plane, with a slice interval of 5 mm. No retakes were allowed because of radiation considerations.

### Reference standard

The study was performed in a transition period from ‘step and shoot transaxial high-resolution CT’ (referred to as ‘transaxial’) to helical CT, and the imaging protocol followed the clinical practice that changed over time. All examinations were performed without intravenous contrast media. Patients were examined by one of the following scanners: Aquilion Prime (Toshiba Medical Systems), Discovery CT750HD, LightSpeed Pro 16, Lightspeed VCT, Optima CT660 (GE Healthcare), Somatom Definition, Somatom Definition Flash, and Somatom Force (Siemens Healthineers). Among the participants, 14/31 (45.2%) were examined by the combination of transaxial technique at baseline and helical at follow-up, and 14/31(45.2%) had helical scans at both occasions. Another 2/31(6.5%) had transaxial series at both occasions, and one had helical CT at baseline and transaxial at follow-up. Slice thickness was 1–1.25 mm in 60/62(96.8%) of the inspiratory scans, and the remaining two were performed with an ultra-low dose helical protocol with 5 mm thick section images in three planes. Additionally, expiratory scans were performed.

### Pulmonary function tests

A PFT was scheduled in conjunction to imaging in a clinical stable period. Absolut values and % of predicted of forced expiratory volume during the first second (FEV1) and forced vital capacity (FVC) were estimated by the method recommended by the Global Lung Function Initiative,^(29)^ and total lung capacity (TLC) and residual volume (RV) according to Hedenström *et al*.^(30,31)^

### Observational study

All images were anonymised, and no clinical information was available to the observers, nor were the observers aware of whether the examinations were from baseline or follow-up. Images were displayed in a randomised order in a room with low ambient light, on a high-resolution medical-grade flat-panel. The observers were able to adjust window width and window level and use the pan/zoom functions.

### Reference CT

Extent of pathological changes on inspiratory and expiratory scans was quantified according to the CF–CT method described by Brody *et al*.^(32)^ Each lobe is scored regarding the extent of disease: extent and size of bronchiectasis (0–72), airway wall thickening/peribronchial thickening (0–54), mucus plugging (0–36) and parenchymal changes such as consolidation, groundglass opacities and cysts (0–54). Hyperinflation is assessed by the extent and distribution of airtrapping on expiratory scan (0–18). One observer employed at a centre with expert knowledge in CF–CT scoring^(33)^ independently and randomly scored all cases. A re-scoring of 25 of the cases followed scoring of the 62 examinations from baseline and follow-up. A second observer at the same institution performed scoring of the same subsample of 25 cases.

### DTS

Extent of pathology was quantified according to a designated tomosynthesis scoring system for CF, which includes the coronal images of DTS and the two-view CXR. The lungs are assessed regarding extent of bronchial wall thickening, atelectasis/consolidation, bronchiectasis (number and appearance) and mucus plugging (large and small), quantified with a score between 0 and 4. The degree of overinflation is assessed on frontal and lateral CXR with a score between 0 and 4. The resulting total score lies between 0 and 100. (KVS) (Observer 1)^(26)^; a paediatric radiologist with 15 years’ experience in paediatric chest imaging including CF, and 10 years’ experience with DTS randomly scored the 62 examinations from baseline and follow-up using the VvS scoring system. All examinations were re-scored 4 weeks after the first session in a new, randomised order. A second reader RRN (Observer 2), a thoracic radiologist with expertise in imaging of CF, with 30 years’ experience in radiology and 9 years’ experience with DTS scored the DTS images from the same randomly selected participants as for CT (*n* = 25).

### Statistical analysis

Scores were reported as absolute values and standardised to percentage of maximum. Statistical calculations were performed with the IBM SPSS Statistics Version 24 software. Intermodality correlation for percentage of total score and subscores was calculated by Spearman’s rank correlation coefficient, where a value ≥0.8 suggests a very strong correlation. The inter-rater reliability for the subjective judgement of extent of disease was analysed with Krippendorff’s alpha (*α*)^(33,34)^ with 95% confidence interval (CI), using SPSS and a macro available at www.afhayes.com/spss-sas-and-mplus-macros-and-code.html. A Krippenfdorff’s *α*-value of 1 indicate perfect reliability, *α* > 0.8 is considered as strong, *α* < 0.67 is low, and *α* = 0 indicates absence of reliability. The cut-off for significant *P* value was adjusted for multiple comparisons according to the Bonferroni method.^(35)^

### Estimation of radiation dose

Radiation exposure for DTS was estimated for a 70 kg standard patient. The dose-area product (DAP) of the DTS examination was calculated from the tube current and DAP value in the digital imaging and communications in medicine header of the frontal scout by the validated method developed by Bath *et al*.,^(36)^ and effective dose was estimated by multiplying the DAP by the conversion factor 0.26 mSvGy^−1^ cm^-2.(^^37^^)^ Effective dose for CT was estimated by multiplying the dose-length product by the conversion factor for chest of 0.017 mSvGy^−1^ cm^−1^, as recommended by European Guidelines.^(38)^

## RESULTS

A total of 186 examinations (DTS, CT and PFT at baseline and follow-up of 31 patients) constituted the material of this study. DTS and CT were performed on the same day. PFT was performed at the same day or ± 1 day as imaging in 91.9% of the cases; patients with acute exacerbations at the time of follow-up were re-scheduled. Participant characteristics at baseline and changes at follow-up are presented in Table 1. The median time between baseline and follow-up examination was 3.0 years (range 2.9–3.2). The median effective dose at baseline was 0.14 mSv (range 0.10–0.16 mSv) for DTS and 2.68 mSv (range 0.54–10.66 mSv) for CT. The follow-up examinations had a median effective dose of 0.13 mSv (range 0.09–0.16 mSv) for DTS and 1.42 mSv (range 0.19–4.98 mSv) for CT.

**Table 1:** Participant characteristics.

	Number (%)	
Women	15 (48.4)	
	Baseline	Follow-up
	Median (range)	Median (range)
Age, years	27.1 (19.8–55.5)	31.1 (22.8–58.5)
Weight, kg	74.0 (45.5–87.6)	68.0 (46.0–89.3)
BMI, kg/m^2^	22.6 (19.1–30.9)	23.0 (18.9–28.2)
TLC, litres	6.4 (4.5–9.2)	6.5 (4.6–9.6)
FEV1, litres	2.9 (1.2–5.3)	2.9 (1.2–5.7)
FVC, litres	4.4 (2.9–6.7)	4.4 (2.5–7.1)
RV, litres	1.9 (1.0–4.5)	2.1 (1.1–5.0)

### Quantification of extent of disease

**Figure 3:**
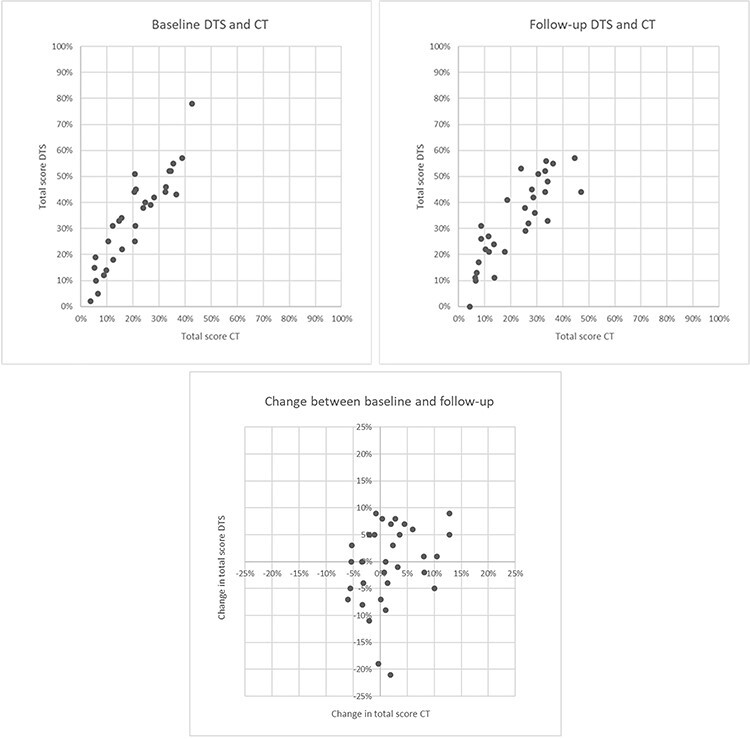
Scatter plot of percentage of maximal score at baseline and follow-up, and change in percentage of total score for CT and DTS.

All CT examinations were assessed as scorable. Two DTS examinations were assessed as non-assessable by Observer 1. Total score for both modalities as well as PFT was overall stable in the study period. Absolute score and PFT (% of predicted) at baseline and change at follow-up is presented in Table 2. Percentage of maximal total score and subscore for baseline and follow-up, and change in percentage of score is presented in Figure 3.

**Table 2:** Total score for each modality at baseline and change in score at follow-up. Lung function parameters at baseline and change at follow-up expressed as percentage of predicted.

	Baseline	Change at follow-up
	Median (range)	Median (range)
Total score CT	50.5 (9.0–103.8)	2.5 (−14.5 to 31.2)
Total score DTS	38 (2–78)	0 (−21 to 9)
FEV1, % of predicted	81 (27–119)	−4 (−19 to 11)
TLC, % of predicted	104 (85–125)	−1 (−21 to 9)
FVC, % of predicted	99 (72–125)	−2 (−15 to 14)
RV, % of predicted	134 (59–219)	12 (−67 to 224)

The Spearman’s rank correlation coefficient between total score for DTS and CT was 0.89 at baseline and 0.86 at follow-up (*P* < 0.01), corresponding values for bronchiectasis were 0.82–0.92 (*P* < 0.01) and for bronchial wall thickening 0.82–0.87 (*P* < 0.01). Additional *r* values are presented in Table 3.

**Table 3:** Spearman’s correlation coefficient for intermodality correlaton for total score and subscore. All scores compared as percentage of maximum. *P* values are adjusted for repeated comparisons ^*^^*^P ≤ 0.01, ^*^^*^^*^P ≤ 0.001.

	DTS-CT baseline	DTS-CT follow-up
Total score	0.89^*^^*^^*^	0.86^*^^*^^*^
Bronchiectasis	0.92^*^^*^^*^	0.82^*^^*^^*^
Bronchial wall thickening	0.87^*^^*^^*^	0.82^*^^*^^*^
Mucus plugging	0.86^*^^*^^*^	0.72^*^^*^^*^
Parenchymal lesions	0.68^*^^*^	0.50^*^^*^
Overinflation	0.40	0.36

**Figure 4:**
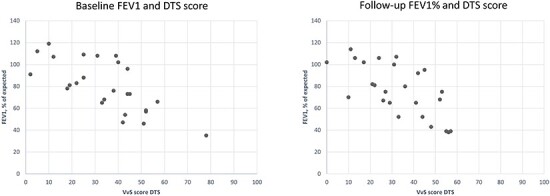
Scatter plot of baseline and follow-up FEV1, % of expected and VvS severity score on DTS, *n* = 25.

The Spearman’s rank correlation coefficient between baseline FEV1 (% of expected) and total severity score was 0.66 for CT and 0.71 for DTS, and at follow-up 0.65 for CT and 0.66 for DTS, with *P* values ≤ 0.001 for all estimates. The correlation between change in FEV1 and severity score was non-significant for both CT and DTS with values of 0.13 and 0.34, respectively. Estimates are based on the 25 participants who underwent lung function test the same or 1 day before/after imaging at both occasions. Scatter plot of FEV1 and DTS score at baseline and follow-up is presented in Figure 4. The correlation coefficient regarding changes in total score between DTS and CT was 0.27.

The intraobserver reliability for total score and subscore was good and comparable for DTS and CT, with Krippendorff’s alpha of 0.94 for total score for both DTS and CT (95% CI 0.91–0.96 and 0.94–0.97, respectively). The interobserver variation was considerable for some of the subscores in both DTS and CT, with values around 0. K alpha values for inter- and intraobserver reliability are presented in Table 4. Differences in scores standardised to percentage of maximum are presented in Table 5.

**Table 4:** Krippendorff’s alpha for intra- and interobserver reliability, 95% confidence interval in parenthesis. (A) Total score. (B) Subscores.

A				
Krippendorff’s alpha				
Total score	DTS		CT	
Intraobserver reliability	0.94 (0.91–0.96)		0.94 (0.91–0.97)	
Interobserver reliability	0.83 (0.76–0.89)		0.75(0.65–0.85)	
B				
	Intraobserver reliability		Interobserver reliability	
Subscore	DTS	CT	DTS	CT
Bronchiectasis	0.94 (0.92–0.96)	0.93 (0.89–0.96)	0.93 (0.89–0.96)	0.86 (0.80–0.92)
Bronchial wall thickening	0.73 (0.62–0.82)	0.93 (0.89–0.96)	0.26(−0.01 to 0.51)	0.37 (0.11–0.60)
Mucus plugging	0.87 (0.76–0.93)	0.87 (0.76–0.93)	0.85(0.74–0.93)	−0.01(−0.30 to 0.26)
Parenchymal lesions	0.79 (0.69–0.88)	0.81 (0.69–0.90)	0.35(−0.07 to 0.70)	0.85(0.75–0.92)
Overinflation	0.80 (0.71–0.89)	0.62 (0.44–0.77)	0.32(−0.16 to 0.72)	−0.06(−0.51 to 0.32)

**Table 5:** Difference in score for repeated observations.

Difference in score (% av maximum), median (range)				
	Intraobserver differences		Interobserver differences	
	DTS	CT	DTS	CT
Total score	4 (−4 to 14)	−0.0 (−9 to 6)	−7 (−14 to 25)	5 (−12 to 19)
Bronchiectasis	3 (−10 to 15)	−1 (−12 to 9)	2 (−25 to 19)	2 (−21 to 23)
Bronchial wall thickening	12 (−25 to 50)	−2 (−9 to 7)	37 (−13 to 75)	15 (−11 to 41)
Mucus plugging	3 (−10 to 15)	−3 (−19 to 8)	0 (−34 to 15)	25 (−6 to 36)
Parenchymal lesions	0 (−13 to 13)	0 (−6 to 4)	−6 (−25 to 7)	0 (−7 to 4)
Overinflation	0 (−25 to 25)	7 (−44 to 33)	0 (−25 to 50)	−19 (−78 to −15)

## DISCUSSION

This study is the first to compare DTS and CT in quantification of structural lung disease in CF, and results show very strong and statistically significant correlation between total score on CT and DTS, with Spearman’s rank correlation coefficients between 0.82 and 0.89 (*P* < 0.01). One could speculate that yearly DTS could be a better alternative than annual CXR and biannual/triannual CT, with the possibility for earlier identification of more subtle changes that are not visible in CXR, at lower total radiation exposure.

### Intermodality correlation

There are currently no available published studies comparing CT and DTS regarding quantification of structural lung disease in patients with CF. The correlation between the subscores for bronchiectasis and bronchial wall thickening in CT and DTS, which are considered as the most important structural finding in CF,^(16)^ was very strong and statistically significant at the 0.01 level. The lowest agreement was seen for the subscore of airtrapping/hyperinflation, and a contributing factor is differences between modalities and their scoring systems. Airtrapping is a specific pattern defined as ‘parenchymal areas with less than normal increase in attenuation and lack of volume reduction’ as seen on expiration CT scans,^(39)^ and the CF–CT scoring involves scoring of both extent as percentage of lobe, as well as the distribution as either subsegmental or segmental or larger. These changes are not adequately depicted in DTS, and hyperinflation is assessed on inspiratory images as an increase in lung volume, with typical signs such as barrel-shaped chest and flattened diaphragm silhouettes. Another factor is differences in the scale steps for the subscore of hyperinflation; the CF–CT scoring system has a maximal score of 27, and tomosynthesis 4. The correlation for parenchymal score was lower than for mucus plugging, bronchial wall thickening and bronchiectasis, which can be explained by the inferior depth resolution in DTS compared to CT, which makes it more difficult to estimate the precise distribution of parenchymal opacities.^(9)^

### Inter- and intraobserver reliability

Inter- and intraobserver reliability for total score was good and comparable for both modalities, as well as the intraobserver reliability for subscores. The interobserver analysis of subscores showed good reliability for bronchiectasis and mucus plugging in DTS, which are considered important structural changes in CF;^(40)^ however, results from the remaining subscores showed considerable variations between observers, which was also the case for subscores for airtrapping and mucus plugging in CT. A reason for this could be that the bronchial tree can be difficult to follow in transaxial step and shoot CT series.

### Detection of changes over time

The correlation between change in FEV1 and severity score, as well as change in total score between DTS and CT was poor and non-significant with values between 0.13 and 0.34.

This study involves imaging in clinically stable patients, PFT values show small changes in the 3 year period between baseline and follow-up, which is similar to results from 14 732 patients in the European Cystic Fibrosis Society Patient Registry database;^(41)^ however, the actual values are difficult to compare because of differences in reference method. Previous studies have found both weak and strong correlation between changes in CT and PFT.^(17,40,42)^ The current study shows weak correlation for changes in FEV1 for both CT and DTS; however, the small changes and limited number of participants in the current study impairs the ability to investigate whether there is a correlation between changes in imaging severity score and PFT. The weak correlation regarding changes in total score between DTS and CT can be influenced by the study setup with individually and randomly evaluated baseline and follow-up examinations, and not side-by-side as in clinical practice.

### Study limitations

The results from this study are based on modality specific scoring systems and observers with expert knowledge of each scoring system. Applying the same scoring system for all modalities would reduce the effect of differences in definitions, scale steps and percentage of subscore in relation to total score. This would, however, be problematic because of the large differences in depiction of pathology, such as groundglass opacities on CT that are difficult to detect on DTS.^(43)^ Having the same observers for all modalities could increase the intermodality correlation even further, but this would, in the current study lead to less experienced observers for one of the modalities. Further, a multicentre study with more participants, a longer study period, and the use of an automated or semi-automated scoring system with higher sensitivity to early structural changes, such as the PRAGMA score^(44)^ that requires helical scans, could be of value in evaluation of small changes in the extent of disease over time.

In conclusion, this study indicates strong correlation between DTS and CT regarding quantification of structural lung disease in CF, with comparable inter- and intraobserver reliability. DTS might be an easy accessible, low dose alternative to standard CT in imaging of patients with CF.

## FUNDING SOURCES

This work was supported by grants from the Swedish state under the agreement between the Swedish government and the county councils, the ALF-agreement (ALFGBG-718111), the Swedish Research Council (2013/3477) and Department of Radiology, Oslo University Hospital, Norway.
